# Robotic Table and Serious Games for Integrative Rehabilitation in the Early Poststroke Phase: Two Case Reports

**DOI:** 10.2196/26990

**Published:** 2022-04-13

**Authors:** Grigore Burdea, Nam Kim, Kevin Polistico, Ashwin Kadaru, Namrata Grampurohit, Jasdeep Hundal, Simcha Pollack

**Affiliations:** 1 Corporate Laboratories Bright Cloud International Corp North Brunswick, NJ United States; 2 Electrical and Computer Engineering Department Rutgers-The State University of New Jersey Piscataway, NJ United States; 3 Department of Occupational Therapy Thomas Jefferson University Philadelphia, PA United States; 4 Hundal Neuropsychology Group Hillsborough, NJ United States; 5 Robert Wood Johnson Medical School Rutgers-The State University of New Jersey Department of Neurology New Brunswick, NJ United States; 6 Computer Information Systems and Decision Sciences St John's University New York City, NY United States

**Keywords:** subacute stroke, virtual reality, gamification, therapeutic game controller, integrative rehabilitation, BrightArm Duo, BrightArm Compact, upper extremity, cognition, depression

## Abstract

**Background:**

BrightArm Compact is a new rehabilitation system for the upper extremities. It provides bimanual training with gradated gravity loading and mediates interactions with cognitively challenging serious games.

**Objective:**

The aim of this study is to design and test a robotic rehabilitation table–based virtual rehabilitation system for functional impact of the integrative training in the early poststroke phase.

**Methods:**

A new robotic rehabilitation table, controllers, and adaptive games were developed. The 2 participants underwent 12 experimental sessions in addition to the standard of care. Standardized measures of upper extremity function (primary outcome), depression, and cognition were administered before and after the intervention. Nonstandardized measures included game variables and subjective evaluations.

**Results:**

The 2 case study participants attained high total arm repetitions per session (504 and 957) and achieved high grasp and finger-extension counts. Training intensity contributed to marked improvements in affected shoulder strength (225% and 100% increase), grasp strength (27% and 16% increase), and pinch strength (31% and 15% increase). The shoulder flexion range increased by 17% and 18% and elbow supination range by 75% and 58%. Improvements in motor function were at or above minimal clinically important difference for the Fugl-Meyer Assessment (11 and 10 points), Chedoke Arm and Hand Activity Inventory (11 and 14 points), and Upper Extremity Functional Index (19 and 23 points). Cognitive and emotive outcomes were mixed. Subjective rating by participants and training therapists were positive (average 4, SD 0.22, on a 5-point Likert scale).

**Conclusions:**

The design of the robotic rehabilitation table was tested on 2 participants in the early poststroke phase, and results are encouraging for upper extremity functional gains and technology acceptance.

**Trial Registration:**

ClinicalTrials.gov NCT04252170; https://clinicaltrials.gov/ct2/show/NCT04252170

## Introduction

### Background

Upper extremity (UE) functional deficits after stroke include reduced range of movement, muscle weakness, low tone, and tremors [1,2]. These motor limitations can be compounded by deficits affecting major cognitive domains of attention, processing speed, executive functioning, memory, and language. Cognitive impairments due to stroke in turn can affect the reacquisition of tasks and new learning. Thus, the combined motor and cognitive deficits adversely affect speed of recovery [3] and the regaining of independence in activities of daily living (ADLs) [4]. The rehabilitation after stroke needs to be integrative, targeting motor UE function *as well as* cognitive functioning. In many health care models, inpatient therapy is limited, expensive, and involves multiple professionals. The ideal training option is leveraging technology for rehabilitation at a single point of care for optimal results and reduced costs.

A high number of task-oriented UE repetitions are needed during therapy to induce the neural rewiring needed to regain function. Brain plasticity is at its peak in the first 6 months after a stroke [5,6]. High-intensity training, meaning many repetitions per minute, is not sufficient by itself. Equally important is the adaptability of the training to individual differences in deficits to improve outcomes and maintain the patient’s motivation.

Technologies increasingly relied upon to meet poststroke-rehabilitation needs include robotic and virtual reality (VR)-based training systems [7]. Both types of systems are popular because they ensure the needed intensity, motivation, and customization. Rehabilitation robots can induce a high number of repetitions and serve as motion guides to improve motor control of the arm during reaching movements [8]. Robots can also assist in UE strengthening [9] in instances where voluntary movement is resisted. However, robotic rehabilitation exoskeletons that wrap around the arm pose safety concerns because of actuators located close to the trained upper limb [10]. The exoskeletons thus require constant supervision and skilled providers to assist in donning and doffing. Robotic systems can be optimized to harness the benefits, reduce skilled supervision, and avoid undesired strain to the UE during training [10]. A promising alternative is a robotic table that automatically adapts for UE training in individuals with stroke.

Bilateral UE training has many advantages over the standard of care (SOC). SOC typically involves unilateral training focused on the affected arm and hand. Advantages of bilateral training include more neural rewiring, strengthening the less-affected UE [11], and ability to train at higher cognitive levels during integrative rehabilitation [12]. However, bilateral robotic rehabilitation using currently available technology is cost prohibitive and requires space, especially in home and community settings [13]. The rehabilitation field needs passive and safe technology (without actuators acting on the trained limbs) to allow bilateral training on a single low-cost and compact system.

VR therapeutic games induce a high number of arm repetitions and are enjoyable. Game-based motor therapy for stroke offers significantly more training [14-17]. Because of the engaging nature of video games, it is easier to alleviate learned disuse and boredom and to induce the number of UE repetitions beneficial to neural recovery after stroke [18]. Moreover, game-based therapy has been widely used in stroke rehabilitation to boost patient motivation, increase exercise intensity, and provide the means to measure objective session-specific outcomes in a quantifiable way [19]. Therapeutic games can be paired with a safe robotic system to amplify the benefits of both forms of rehabilitation training when used together.

### Related Work

**Figure 1:**
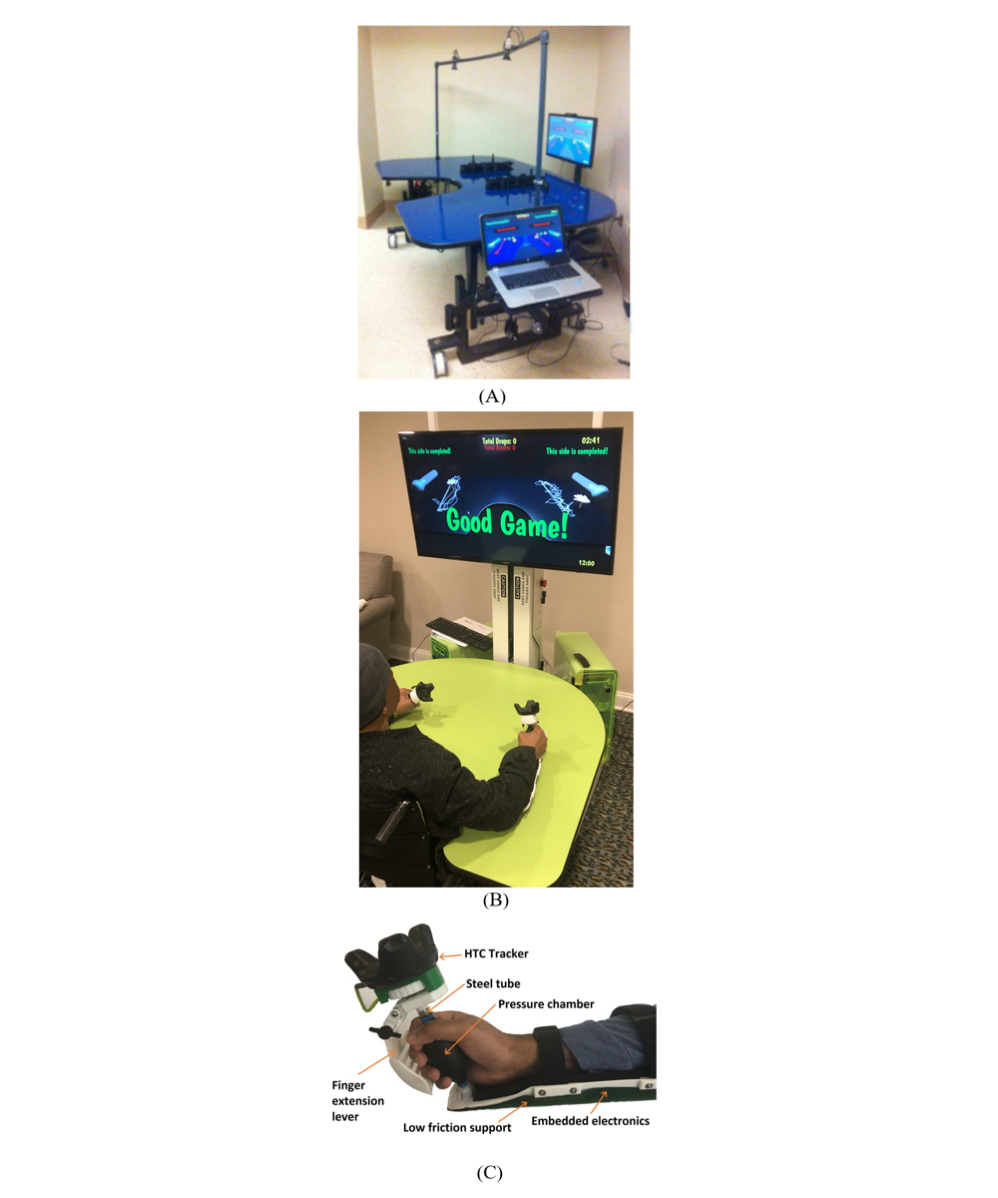
Robotic rehabilitation tables and controller: (A) the BrightArm Duo system, (B) the BrightArm Compact system training case 1, and (C) the BrightBrainer Grasp therapeutic game controller. Reprinted by permission of Bright Cloud International Corp.

A precursor to the robotic system reported here was the BrightArm Duo robotic table (Figure 1A). It used a low-friction motorized table to help forward arm reach, assisted supported reaching by tilting its distal side down, and resisted reaching by tilting the table surface up. Arms were placed in low-friction forearm supports with embedded infrared (IR) light-emitting diodes. The arm supports could slide on the rehabilitation table and were tracked by a pair of overhead IR cameras. The cameras communicated with a PC running the table actuators as well as its therapeutic games. These games were presented on a large display in front of the patient and could be played during unilateral or bilateral rehabilitation (Figure 1A).

For all its advances, BrightArm Duo had shortcomings too. It was a large system with complex controls, owing to its 2 table-lifting and 2 table-tilting actuators. Furthermore, the flat bottom of the forearm supports made it impractical to train pronation and supination while supported on the table. Moreover, it was not possible to train finger extension, which is key to grasping objects and critical to increasing ADL independence. Thus, the BrightArm Duo did not address a missing element in the field of rehabilitation technology [11,20,21], namely the lack of an integrative system of training finger extension, forearm pronation and supination, hand grasping, bilateral movements, and engaging cognitive rehabilitation.

Our novel *BrightArm Compact* (BAC) system addresses the aforementioned missing element. The redesign of the system and a rigorous evaluation process allowed us to embed new and improved features. The modulation of gravity bearing can support the weaker side and enable UE strengthening [20,21]. The table can facilitate integrative motor and cognitive training when coupled with challenging therapeutic games [22]. However, the testing of the BAC system with individuals with stroke (preferably in the early stages after stroke) is essential to measure the impact on function. Findings from the preliminary evaluation can then inform larger studies and advance the field of rehabilitation technology. This is the motivation behind this study.

This paper presents the first clinical study of the next-generation BAC rehabilitation robotic table. It consists of 2 case studies who trained on the BAC system during the early subacute phase after stroke.

The following research questions were addressed:

1. Primary: How does bilateral training with the integrative rehabilitation system impact UE function?
2. Secondary: Does the integrative rehabilitation system have an impact on the secondary outcomes of cognition, emotion, and game performance?
3. What is the perception (positive or negative) of the participants and the therapists who used the new system at an inpatient clinic?

Technology details of the robotic table and its integrative therapeutic games are presented first. The recruitment procedures, training protocol, and outcome measures are described subsequently, followed by the case-specific results and the *Discussion* and *Conclusions* sections.

## Methods

### The BAC Rehabilitation Table

The BAC rehabilitation table had a streamlined design using a single linear actuator for lifting movement and a second linear actuator for work-surface tilting. The first linear actuator adjusted the table to the patient’s height such that the arms could be supported without shoulder discomfort (Figure 1B). The second actuator was used to adjust the work-surface tilt angle between 20° uptilt and –15° downtilt. In this design, 0° corresponded to a horizontal table. Both actuators were housed in a central column that also supported a large television displaying therapeutic games. Because of its more compact design, the BAC system’s overall footprint was 45% smaller than that of the Duo precursor, while still allowing full bilateral supported arm reach.

Arm lifting off the table was possible because, unlike in the case of other rehabilitation robots, no actuators directly pushed on the UEs. The added advantages were increased freedom of movement and enhanced patient safety. Another component of the BAC safety mechanism was an array of IR illuminator strips located on the underside of the work surface. The IR sensing strips were arranged to detect a patient’s presence while seated at the table in a chair or wheelchair. Proximity with the patient’s knees was also detected, in which case the table motion for lifting or tilting was momentarily paused. Another safety measure was a mechanism designed to detect imminent collision between the table underside and the top of a wheelchair wheel. Such collisions could occur during the upward tilting of the table, depending on the height and type of wheelchair. Finally, a pair of emergency power shutoff switches were mounted on either side of the central tower assembly. It was easy to reach the location of the switches regardless of which side of the patient the therapist stood to assist with right- or left-arm training.

Rehabilitation with the BAC robotic table was facilitated by a pair of BrightBrainer Grasp (BBG) therapeutic game controllers which our group developed [23]. As shown in Figure 1C, the BBG used an HTC tracker (HTC Corp) to measure hand movement in 6 degrees of freedom. The HTC tracker should not be confused with the VIVE controller (HTC Corp), which was not used with the BAC system. The HTC tracker’s position and orientation were measured in real time with the aid of a pair of VIVE IR illuminators (or *lighthouses*). The 2 VIVE lighthouses were located on either side of the central actuating column.

The BBG controller had a rubber pear and a pressure sensor to measure grasp strength and a rotating mechanical lever to measure finger extension. The underside of the controller was curved to allow supported pronation and supination and covered in a low-friction material to facilitate supported arm reach. The same curved shell housed electronics and batteries as well as a wireless transmitter for bidirectional communication with a PC running the therapeutic games.

Therapeutic game controllers must be simple to use to avoid taxing the limited resources of individuals with a disability and avoid increasing the setup time for their therapists and families. Furthermore, the controller’s shape must accommodate hands of various sizes and functional levels. In the BBG game controllers, these general principles were applied to detect finger extension and grasping. The curved shape of the mechanical lever maintained positive contact with the outer side of the patient’s hand to detect extension regardless of which finger or fingers pushed it outward. Conversely, grasping was detected regardless of which finger or fingers flexed around the BBG rubber pear. Additional details of the BAC design and its usability evaluation study can be found in Burdea et al [24], whereas the clinical results in individuals in the chronic phase after stroke using the BBG can be found in Burdea et al [25].

A baselining process enabled adaptation to a particular patient’s motor function level. The baseline mapped different motor functions of the weak and strong UEs to the normal functions of the left and right avatars in the therapeutic games. The baseline was captured for grasp, finger extension, arm pronation, arm supination, vertical reach, and horizontal reach. Vertical reach and horizontal reach baselines were recorded for 1 arm at a time, as previously described for the BrightArm Duo [20]. The other baselines were captured simultaneously for both UEs to reduce overall system setup time.

During the finger-extension baseline, the patient watched a scene showing 2 simplified controllers moving their respective mechanical levers in response. Simultaneously, 2 vertical tubes were filled with color to visualize the magnitude of the extension angle of the corresponding hand. The grasp baseline scene was similar, and the amount of color in the vertical tubes was proportional to each hand’s grasping strength. The baseline process was repeated 3 times, and the net value was calculated after subtracting the residual force.

Baselines were subsequently used to determine gains between UE movements and those of the avatars controlled in a game. The impaired UE limited reach was mapped to the full extent of VR scenes. Presenting fully functional avatars was aimed at making the games winnable to reduce depression [26]. Only a fraction of the maximal finger-extension range and maximal grasping force were used to control the game. The use of fractional values reduced fatigue and discomfort during prolonged virtual rehabilitation sessions. The baseline was used to determine thresholds for hand-avatar flexion or extension. Once a threshold was exceeded in the extension direction, the game software commanded a hand avatar to open fully. Similarly, once a grasping force threshold had been exceeded, the hand avatar was commanded to close fully.

**Figure 2:**
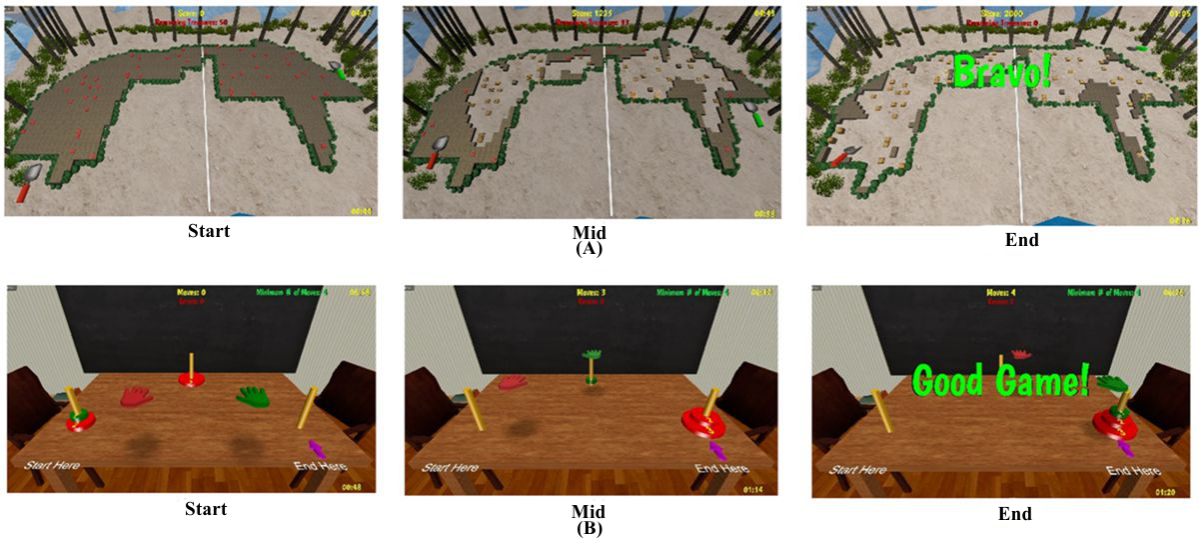
A sample of integrative therapeutic games played during the BrightArm Compact study. Sequence, from left to right, shows game scenes at start, midgame, and end for (A) *Treasure Island* and (B) *Towers of Hanoi 3D*. Reprinted by permission of Bright Cloud International Corp.

What follows is a description of 2 of the therapeutic games used in the BAC rehabilitation system. *Treasure Island* (Figure 2A) was a game training UE endurance, coordination, and short-term visual memory. An island was depicted where treasures were dug out from an area marked with boulders. Patients were required to use a grasp for lowering the shovel avatar into the sand or move the shovel to other locations using the extension. Treasures were given different gold counts, with those closest to the boulder wall (which visualized the horizontal reach baseline) containing more gold. The game ended once all treasures had been found or the allotted time had ended. Lower levels of difficulty had markings on the sand to indicate where treasures were buried, and the weather was calm. There were more treasures at higher levels of difficulty (more repetitions) to be dug out in a shorter time (faster movements), and no markings were present. For even higher levels, sandstorms would cover some of the treasures that had been discovered such that their locations needed to be remembered and more UE movements were elicited to dig them up again.

*Towers of Hanoi 3D* (Figure 2B) was used to train primarily executive function. The game trained decision-making by asking the patient to restack disks of varying diameters from 1 of 3 poles to another while using the third pole as a waypoint. The version of the game for the BBG and BAC required grasping to pick up a disk, reaching to bring that disk above a pole, then extending fingers to release the disk onto that pole. Decision-making was trained by rules requiring that a larger diameter disk could never be placed on top of a smaller one and that disks be handled only by like-colored hand avatars. The smaller disk had the color of one of the hand avatars (eg, red), and the other disks had the color of the other hand avatar (eg, green). The aim was to restack the disks with a minimal number of moves, which depended on the number of disks in the game (eg, restacking 3 disks required a minimum of 7 arm-reach moves, 7 grasps, and 7 finger extensions).

A total of 8 different games were used in this study. Each game had up to 16 levels of difficulty to ensure variety and challenge during BAC training. When a game was repeated in several sessions, its actual difficulty was set automatically, based on a particular patient’s past performance in that game. If the patient failed to finish the game or obtained a low score 2 consecutive times, the difficulty level was reduced by 1 level in the next play. In contrast, if a patient won a game 3 consecutive times, then that game difficulty was increased by 1 level in the next play.

### Recruitment

In early September 2018, 1 BAC system was placed at PowerBack Rehabilitation (Piscataway, New Jersey, United States), an inpatient rehabilitation facility specializing in early subacute recovery stages. The inpatient rehabilitation director (an occupational therapist [OT]) and another licensed OT were trained in the use of the BAC system. Subsequently, 2 cases described here who received SOC at the facility were screened, and both provided informed consent to participate in this study.

Case 1 was a right-handed African American male, 83 years of age, with left arm affected by a hemorrhagic stroke to the right frontal lobe, right inferior thalamus, and right superior cerebellar peduncle. The stroke had occurred 7 weeks before enrollment. He presented with hypertension, atrial fibrillation, and a visual field cut on his left side. Case 1 was taking 10 prescription medications at the time of enrollment (Milk of magnesia, Lisinopril, Dulcolax, Dorzolamide, Allopurinol, Brimonidine tartrate, Pravastatin, Eliquis, Flomax, and Amlodipine). He was able to ambulate 70 feet with a single-point cane, with supervision. The initial Fugl-Meyer UE [27] score was 45 out of 66, indicating mild impairment. He had 12 years of formal education, was a native English speaker, and a retired truck driver.

Case 2 was a left-handed White male, 66 years of age, with affected left UE after a right hemorrhagic stroke (basal ganglia infarct) that occurred 3 weeks before enrollment. He was higher functioning in motor performance than case 1, with an initial Fugl-Meyer UE score of 52 out of 66, indicating mild impairment. Case 2 had anemia, hypertension, and was on 6 medications during this study (Atorvastatin, Calcium, Cyanocobalamin, Midodrine, Polyethylene glycol powder, and Folic Acid). He was able to ambulate 50 feet using a rolling walker independently. Case 2 had 12 years of formal education, was an English speaker, and his previous occupations were painter and landscaper.

### Data Collection Instruments

#### Overview

**Figure 3:**
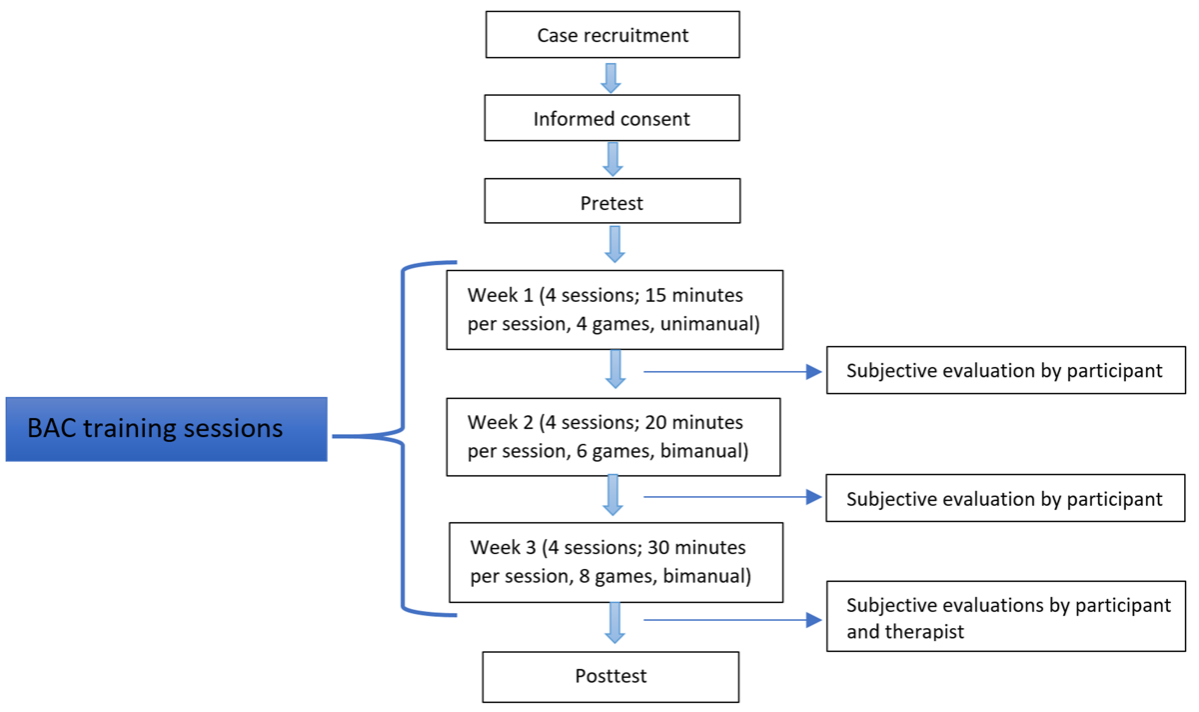
Flowchart diagram of the case study protocol. Reprinted by permission of Bright Cloud International Corp. BAC: BrightArm Compact.

This study followed an ABA protocol, with data collected at baseline or pretest (A), at every training session (B), and at the end of rehabilitation on the experimental system (A; Figure 3).

The pre- and posttraining clinical evaluations measured motor impairment and function, cognitive function, and emotional state. These were assessed using standardized instruments and supplemented by data from nonstandard measures, as described in the next sections.

#### Evaluation of Motor Impairments

Active range of motion was measured using a standard goniometer to determine the active arm’s and fingers’ range of movement on both the impaired and unimpaired sides. Calibrated wrist weights were used to determine shoulder strength when lifting the straight arm in front of the body to a horizontal position (anterior deltoid) and lateral to the body to a horizontal position (lateral deltoid). A mechanical Jamar dynamometer was used to measure grasp strength, and a Jamar pinch gauge was used to assess finger pinch strength. Both instruments have been shown to have adequate reliability and validity for this purpose [28].

#### Function of the UE

This was assessed with (1) the UE subscale of the Fugl-Meyer Assessment with a score ranging from 0 to 66, where 0 is most severely impaired and 66 is normal UE function; (2) the Jebsen Test of Hand Function [29], a timed test of 7 simulated ADLs, each timed from 0 to 180 seconds; (3) the Chedoke Arm and Hand Activity Inventory [30], which measures independence in 9 bimanual ADLs, each scored from 0 to 7. Here, a 0 means the participant needs total assistance in performing a task, whereas a 7 means complete independence in performing it; and (4) the Upper Extremity Functional Index (UEFI) [31], a self-report of independence in 20 ADLs, each scored on a 0-4 scale, where 0 corresponds to inability to perform a task and 4 corresponds to no difficulty at all in performing it. All these measures have been reported to have good psychometric properties for assessing function in stroke.

#### Emotive State

This was measured with the Beck Depression Inventory, Second Edition (BDI-II), as an indication of depression severity, compatible with its reliability and validity for this use [32].

#### Cognitive Function

This was assessed with the Brief Visuospatial Memory Test, Revised [33], for delayed visual memory recall (forms 1 and 2); Hopkins Verbal Learning Test, Revised [34], for delayed verbal memory recall (forms 1 and 2); the Neuropsychological Assessment Battery (NAB) word generation subtest of the executive functioning module [35] for executive function and verbal fluency (forms 1 and 2); the NAB digit span forward and backward test for auditory attention and working, as well as the Dots subtest for visual working memory; Trail Making Test Part A for visual attention and information-processing speed; and Trail Making Test Part B as a measure of executive function and mental flexibility. The psychometric properties of these measures for stroke indicate high reliability and validity [36].

#### Game Performance Data

These consisted of objective measures of therapeutic gameplay performance. Motor domain variables were arm repetitions, grasp and finger-extension repetitions, the intensity of training (as repetitions per minute), and area and shape of arm reach (measured by the BAC system). In the cognitive domain, data stored were game average difficulty level (per session), game average duration, and total cognitive exercise time. This training time was reported for the specific cognitive domains of executive function, attention, and memory. These game data were deidentified, automatically sampled at each experimental session, and uploaded on a Microsoft Azure [37] secure cloud server.

A remote graphing capability was developed to allow researchers to log in to the project portal and remotely review an individual’s game performance data.

#### Subjective Evaluation Custom Forms

These were developed for the participants, and separate, somewhat different forms, were developed for the OTs assisting in their training. The evaluation form to be completed by the participants after stroke, shown in Table 1, had 15 items. Each item used a 5-point Likert rating scale, with 1=least desirable outcome and 5=most desirable outcome. Participants were requested to complete the form at the end of every experimental training week to longitudinally determine changes in rating as games became harder with longer sessions. The participants’ ratings of the system are included in the table as well but will be discussed later.

**Table 1:** Subjective evaluation scores (1=least desirable outcome and 5=most desirable outcome) from 2 case studies. Each participant submitted 1 feedback form per week for 3 weeks^a^.

Item	Participants’ scores										Question average score (SD)^b^
	Case 1^c^					Case 2^d^					
	Week 1	Week 2	Week 3	Average (SD)	Week 1		Week 2	Week 3	Average (SD)		
1. Instructions given to me were useful	4	4	4	4.0 (0.00)	4		4	3	3.7 (0.58)	3.8 (0.41)	
2. The system was easy to use	4	4	2	3.3 (1.15)	4		4	4	4.0 (0.00)	3.7 (0.82)	
3. The game controllers worked the way I wanted them to	4	3	2	3.0 (1.00)	4		3	4	3.7 (0.58)	3.3 (0.82)	
4. It was easy to put the controllers on and take them off	5	4	4	4.3 (0.58)	4		3	4	3.7(0.58)	4.0 (0.63)	
5. The controllers made little noise	5	4	5	4.7 (0.58)	4		4	4	4.0 (0.00)	4.3 (0.52)	
6. The television was a suitable distance away	4	4	4	4.0 (0.00)	4		4	4	4.0 (0.00)	4.0 (0.00)	
7. The games were interesting	4	4	4	4.0 (0.00)	4		4	4	4.0 (0.00)	4.0 (0.00)	
8. I had no muscle pain or discomfort	5	5	4	4.7 (0.58)	4		4	4	4.0 (0.00)	4.3 (0.52)	
9. I was not fatigued by the end of the game therapy session	3	4	3	3.3 (0.58)	4		4	3	3.7 (0.58)	3.5 (0.55)	
10. I was not bored while exercising	4	4	5	4.3 (0.58)	4		4	4	4.0 (0.00)	4.1 (0.41)	
11. The length of game exercising in a day was appropriate	4	3	4	3.7 (0.58)	4		4	4	4.0 (0.00)	3.8 (0.41)	
12. There were few technical problems	3	4	4	3.7 (0.58)	4		3	4	3.7 (0.58)	3.7 (0.52)	
13. I would encourage other patients to use it	4	4	4	4.0 (0.00)	4		5	4	4.3 (0.58)	4.1 (0.41)	
14. I liked the system overall	4	4	4	4.0 (0.00)	4		4	4	4.0 (0.00)	4.0 (0.00)	
15. The controllers were easy to slide along the table	4	4	5	4.3 (0.58)	4		5	5	4.7 (0.58)	4.5 (0.55)	

^a^Reprinted by permission of Bright Cloud International.

^b^Participants’ average score for all questions is 3.97 (SD 0.03).

^c^Participant 1 average score for all questions is 3.95 (SD 0.67).

^d^Participant 2 average score for all questions is 3.95 (SD 0.42).

Table 2 shows the subjective evaluation items for the attending therapists. This involved rating on a similar 5-point Likert scale, but the items used were different from those presented in Table 1. The therapists’ questions were designed to gauge their ability to learn how to use the system, their perceived level of case discomfort, the appropriateness of training intensity on the BAC robotic table, and overall level of satisfaction with the system. The therapists’ ratings of the system are included as well in Table 2.

**Table 2:** Therapist evaluation scores (1=least desirable outcome and 5=most desirable outcome) for the BrightArm Compact system at the completion of the experimental training (session 12)^a^.

Items	Scores			Question average score (SD)^b^
	Therapist 1^c^	Therapist 2^d^		
1. It was easy to learn how to use this system	4	4	4.0 (0.00)	
2. It was easy to show the patient how to use the system	4	2	3.0 (1.41)	
3. It was easy to set up and run the session	4	2	3.0 (1.41)	
4. It was easy to manually enter notes during the session	3	4	3.5 (0.71)	
5. It was easy to put the controller on and take it off	4	4	4.0 (0.00)	
6. The controller provided good grasp training	5	5	5.0 (0.00)	
7. The controller provided good finger-extension training	4	5	4.5 (0.71)	
8. Patients did not appear to experience discomfort during exercises	4	5	4.5 (0.71)	
9. The system reduced amount of OT^e^ assistance needed	4	3	3.5 (0.71)	
10. There were few technical problems using the system	4	2	3.0 (1.41)	
11. The length of exercise was appropriate for the patient	4	4	4.0 (0.00)	
12. The session reports provided useful information	4	4	4.0 (0.00)	
13. The intensity of training was appropriate	5	3	4.0 (1.41)	
14. Overall, I am satisfied with this system	5	4	4.5 (0.71)	

^a^Reprinted by permission of Bright Cloud International.

^b^Therapist’s average score for all questions is 3.89 (SD 0.62).

^c^Therapist 1 average score for all questions is 4.1 (SD 0.53).

^d^Therapist 2 average score for all questions is 3.6 (SD 1.08).

^e^OT: occupational therapist.

### Protocol

Each participant was seated at the BAC system such that the abdominal area touched the inside of the table cutout. Next, the table height was set to ensure comfortable supported movement of the arms, with minimal shoulder discomfort. Subsequent to the vital signs being checked and the game controllers being donned, the therapist instructed the participant to perform arm reach horizontally and vertically, grasp, extend fingers, and finally move the supporting arm in pronation and supination directions. The protocol set week 1 training to be unimanual (unilateral); thus, baselining captured only the affected UE. In weeks 2 and 3, both UEs were baselined and trained. Each session was paused automatically midway to allow the therapist to recheck vital signs. Checking of vital signs was repeated at the end of every session. Sessions could be paused to introduce a rest period in case the participants felt fatigued or experienced pain.

Each case study participant trained every other day, including weekends, and completed 12 sessions over 3 weeks of experimental training. The session duration lengthened progressively, from 15 minutes of exercising in week 1 to 20 minutes in week 2 and 30 minutes of gameplay in week 3. During this period, the participants played 4 different games in week 1, 6 games in week 2, and 8 games in week 3. These game sequences were repeated as needed to complete the prescribed session exercise duration for that week. Game difficulty was preset to easiest level in week 1 and was progressed automatically such that the hardest levels were in week 3. Playing bimanually (using both hands) in weeks 2 and 3 increased physical and cognitive effort requiring hand-eye coordination and split attention.

During each session, the engineer used TeamViewer [38] to remotely access the system. The remote access allowed the engineer to monitor and assist experimental sessions in real time remotely, if needed. Technical issues were addressed in consultation with the therapist, and any required software updates were completed overnight.

Researchers also accessed a password-protected project portal separately. Information stored on this portal was graphed longitudinally to better gauge participants’ progress based on system-generated variables and system-generated rehabilitation session reports. These functionalities were available at any time, regardless of whether a rehabilitation session was in progress.

In addition to the BAC experimental therapy, the 2 cases received physical therapy, occupational therapy, and speech therapy as inpatients at the PowerBack Rehabilitation facility. Each week they received 6-7 sessions of physical therapy lasting for 45 to 60 minutes each, 6-7 sessions of occupational therapy lasting for 45 to 60 minutes each, and 5 sessions of speech therapy lasting for 30 minutes each.

### Ethical Considerations

Initial human participant approval was received from the Western Institutional Review Board (Protocol#20101313; now renamed WCG IRB), and participants provided informed consent.

## Results

### Outcomes

The participants’ game performance progression during the 3 weeks of experimental BAC training is shown in Table 3.

The changes in motor impairment, function, and ADL independence are shown in Table 4, whereas Table 5 shows changes in the participants’ emotive and cognitive functions.

**Figure 4:**
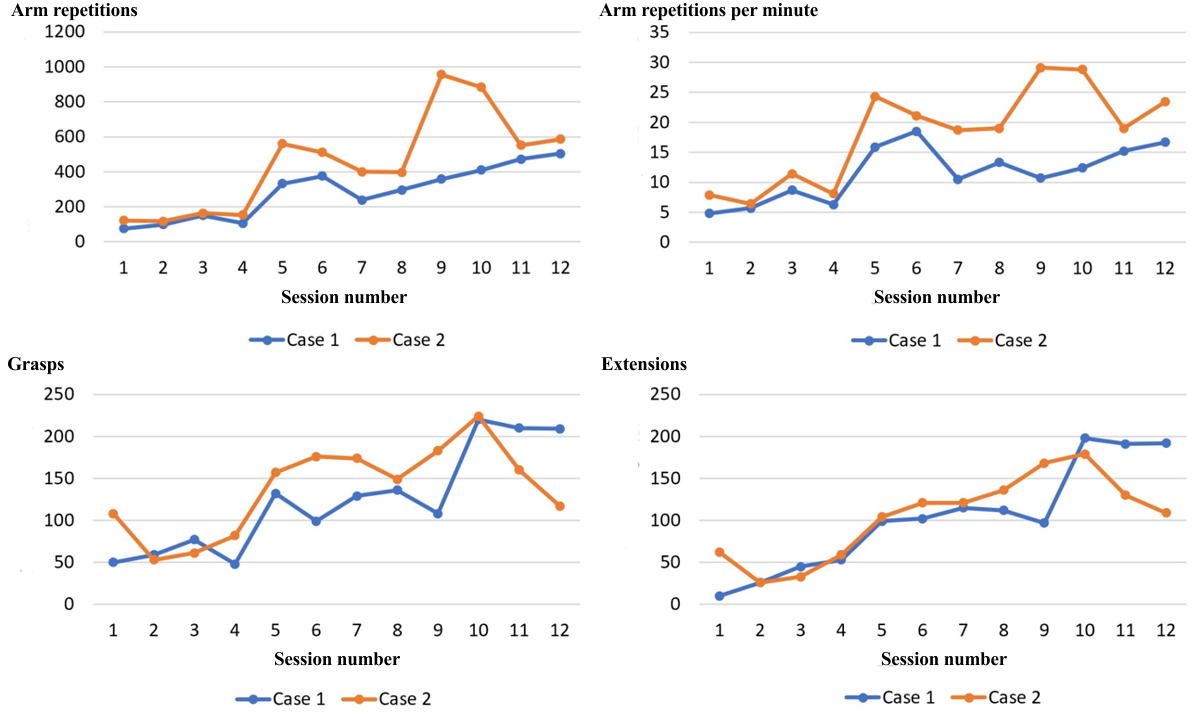
Game performance for the 2 participants early subacute phase after stroke training on the BrightArm Compact robotic rehabilitation table. Reprinted by permission of Bright Cloud International Corp.

**Figure 5:**
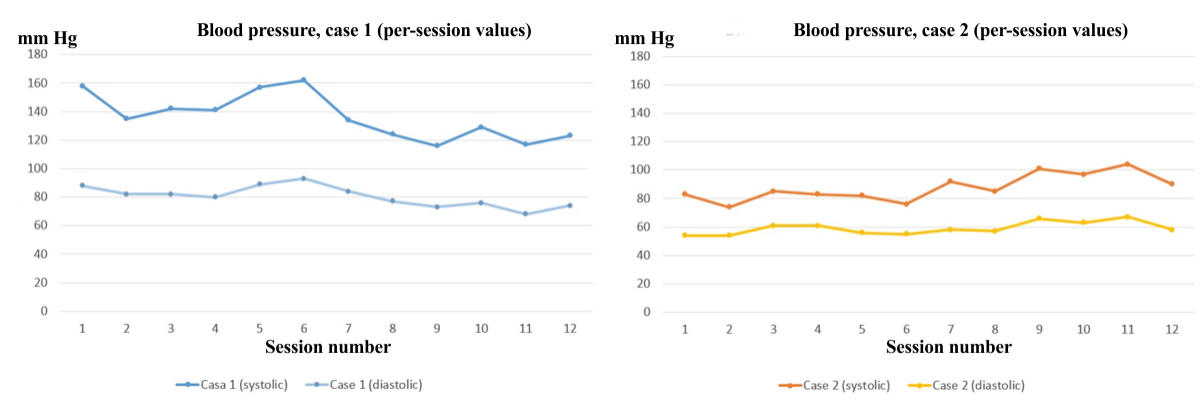
Participants’ blood pressure progression over 12 BrightArm Compact rehabilitation sessions. Reprinted by permission of Bright Cloud International Corp.

The main game performance variables over the 12 experimental sessions are displayed in Figure 4. The systolic and diastolic blood pressure progression over the 3 weeks of experimental training for the 2 cases are represented by graphs in Figure 5.

**Table 3:** Game performance outcomes for the 2 cases over 3 weeks of training with the BrightArm Compact therapeutic game system. Each case’s session 1 game performance and highest one are presented for comparison^a^.

Outcomes		Case 1		Case 2	
		Session 1	Highest	Session 1	Highest
**Games targeting motor training**					
	Session arm repetitions	75	504	122	957
	Repetitions per minute	5	18	8	29
	Session grasps	50	220	108	224
	Grasps per minute	3	7	7	7
	Finger extensions	10	198	62	179
	Extensions per minute	<1	6	4	6
**Games targeting cognitive training**					
	Game average difficulty (per session)	1.5	3.5	1.5	2.9
	Cognitive training time (minutes per session)	16	34	16	33

^a^Reprinted by permission of Bright Cloud International.

**Table 4:** Changes in the cases’ affected upper extremity impairments, function, and independence in activities of daily living over 3 weeks of training with the BrightArm Compact system^a^.

Outcomes			Case 1							Case 2				
			Before the training		After the training		Difference		Before the training			After the training		Difference
**Upper extremity motor impairments**														
	Shoulder strength (anterior deltoid; N^b^)	4.4		11.1		6.7		8.9			20.0		11.1	
	Shoulder strength (lateral deltoid; N)	0		6.7		6.7		6.7			13.3		6.6	
	Grasp strength (N)	194		247		53 (61)^c,d^		96			111		15 (49)^c,d^	
	Three-finger pinch strength (N)	36		47		11		12			14		2	
	Shoulder flexion (°)	100		117		17		118			139		21	
	Shoulder extension (°)	N/A^e^		N/A		N/A		22			24		2	
	Shoulder abduction (°)	92		118		26		110			121		11	
	Shoulder adduction (°)	20		33		13		41			50		9	
	Elbow flexion (°)	120		131		11		127			141		14	
	Elbow extension (°)	–20		–10		10		0			0		0	
	Elbow pronation (°)	45		70		25		63			90		27	
	Elbow supination (°)	40		70		30		57			90		33	
	Thumb MCP^f^ flexion (°)	80		84		4		90			90		0	
	Index finger MCP flexion (°)	78		82		4		90			90		0	
	Middle finger MCP flexion (°)	85		85		0		90			90		0	
	Ring finger MCP flexion (°)	80		87		7		90			90		0	
	Little finger MCP (°)	78		78		0		90			90		0	
**Upper extremity motor function**														
	Fugl-Meyer upper extremity score (maximum 66; higher is better)	45		55		11 (9 to 10)^d^		52			62		10 (9 to 10)^d^	
	Jebsen Test of Hand Function total completion time (seconds; less is better)	147		126		–21 (–20.8)^d^		82			57		–25 (–20.8)^d^	
	Chedoke Arm and Hand Activity Inventory score (maximum 63; higher is better), bimanual	40		51		11 (6.3)^d^		44			58		14 (6.3)^d^	
	Upper Extremity Functional Index 20	32		51		19 (8)^d^		57			80		23 (8)^d^	

^a^Reprinted by permission of Bright Cloud International.

^b^N: newton.

^c^Different minimal clinically important difference values for grasp strength reflect arm dominance versus arm affected. A 0° angle value indicates full extension to a straight arm (for elbow) and a straight hand for finger metacarpophalangeal joints.

^d^Minimal clinically important difference for that measure.

^e^N/A: not applicable.

^f^MCP: metacarpophalangeal joint

**Table 5:** Emotive and cognitive outcomes of the cases who were in the early subacute phase after stroke^a^.

Categories and assessment						Case 1							Case 2			
						Before the training			After the training			Before the training				After the training
**Emotive**																
	**Mood and personality**															
		Beck Depression Inventory, Second Edition			4(↓^b^ is better)			0			5				8 (60%↑^c^)	
**Cognitive**																
	**Attention and processing speed**															
		**Neuropsychological Assessment Battery**														
			Digits forward	10			6 (40%↓)			5				10 (100%↑)		
			Longest digit span forward	7			5 (40%↓)			5				7 (40%↑)		
			Digits backward	3			4 (33%↑)			3				4 (33%↑)		
			Longest digit span backward	2			4 (100%↑)			4				4 (0%)		
			Dots	3			1 (66%↓)			2				5 (150%↑)		
		**Trail Making Test A**														
			Trails A	189			>300 I (59%↑)			72				82 (14%↑)		
	**Verbal memory**															
		**Hopkins Verbal Learning Test–Revised**														
			Trials 1 to 3	18			19 (5%↑)			12				11 (40% ↓)		
			Delayed recall	5			4 (20%↓)			0				0		
			Recognition discrimination score	7			12 (71%↑)			4				6 (50%↑)		
	**Visual memory**															
		**Brief Visuospatial Memory Test, form 2**														
			Trials 1 to 3	5			6 (20%↑)			5				4 (40%↓)		
			Delayed recall	2			3 (50%↑)			3				2 (40%↓)		
			Recognition discrimination score	2			2 (0%)			1				3 (200%↑)		
	**Orientation**															
		**Neuropsychological Assessment Battery, form 2**														
			Person	14			14 (0%)			14				14 (0%)		
			Time	8			7 (12%↓)			6				5 (40%↓)		
			Place	3			3 (0%)			3				4 (33%↑)		
	**Executive functioning**															
		Trail Making Test B			>300 (D/C)^d^			>300 (D/C)			>300 (D/C)				194 (35%↓)	
		**Neuropsychological Assessment Battery**														
			Word generation, total number of words	5			4 (20%↓)			4				5 (25%↑)		
			Word generation, total number of perseverations	0			0 (0%)			1				0 (100%↓)		

^a^Reprinted by permission of Bright Cloud International.

^b^Arrows pointing down symbolize a decrease in the respective variables post intervention.

^c^Arrows pointing up symbolize an increase in the respective variables post intervention.

^d^D/C: test discontinued after exceeding 300 seconds maximum allowed time.

### Case 1

#### Game Performance Outcomes

As seen in Figure 4, case 1’s first session on the BAC system had only 75 movement repetitions of the affected left arm. However, he attained 504 total (right+left) arm repetitions by his last experimental training session, which was played bimanually. The number of grasps grew from 50 in the first session to a maximum of 220 grasps for left and right hands combined. Similarly, the number of finger extensions grew from 10 for the hand in the first session to a high of 198 combined left- and right-hand extensions per session in session 10.

The aforementioned increases in repetitions were due in part to longer sessions, more challenging game levels, and progressing from unilateral training in week 1 to bilateral training in weeks 2 and 3. A measure that normalizes for session duration is the training intensity because it reports repetitions per minute. For case 1, training intensity grew by 360% for arm movements per minute, 233% for grasps per minute, and 600% for finger extensions per minute.

As the game-based rehabilitation was integrative, it incorporated cognitive training. As a measure of cognitive task complexity (cognitive load), the game difficulty increased for case 1 from an average level of difficulty of 1.5 in the first session to a high of 3.5 average game difficulty per session during the 3-week training. The number of cognitive training minutes per session, indicating cognitive endurance, more than doubled, from 16 minutes in session 1 to 34 minutes of cognitive training per session during the 3-week training.

#### Motor Impairment

The motor impairment before and after the training for case 1 is shown in Table 4 for the affected nondominant left arm. Shoulder strength increased by 6.7 N for the anterior and lateral deltoid muscles, indicating the efficacy of the gravity-modulating rehabilitation table. Hand grasp strength increased from 194 N before the training to 247 N after the training. This gain of 53 N was below the 61 N minimal clinically important difference (MCID) in grasp strength increase in the nondominant arm for populations in the subacute phase after stroke. The 3-finger pinch strength went from 36 N before the training to 47 N after the training (31% improvement). There were increases in the affected arm’s active range of movement, most notable for shoulder abduction (26° range increase), elbow pronation (25° increase), and supination (30° increase). Range of movement when flexing fingers improved slightly from before the training to after the training for the metacarpal joint. His finger-extension range did not improve because he had normal extension before the training.

#### Motor UE Function

Case 1’s UE function improved, with his Fugl-Meyer score increasing 11 points, above the MCID of 9-10 points for individuals in the subacute phase after stroke [39]. He became faster in simulated ADLs, with a 21-second reduction in the time it took to complete the Jebsen Test of Hand Function (MCID –20.8 seconds for chronic stage [40]; however, no value exists for patients in the subacute phase after stroke). Case 1’s independence in bimanual ADLs, measured by his score on the Chedoke Arm and Hand Activity Inventory, increased by 11 points, well above the MCID of 6.3 points for this measure [30]. On the standardized UEFI 20-question self-report, case 1’s score increased by 19 points, more than double the corresponding MCID of 8 points [31].

#### Emotive and Cognitive Outcomes

Before the training, case 1’s BDI-II score of 4 indicated minimal depression. After the training, his score was 0, indicating normal mood. As seen in Table 5, case 1’s neurocognitive evaluation showed a negative gain in his executive function (NAB word generation raw score decreased from 5 before the training to 4 after the training). This was matched by worse performance in Trail Making Test Part A from 189 seconds before the training to >300 seconds after the training (unable to perform), indicating diminishing attention and processing speed. However, there was improvement in 2 of the 3 scores of visual memory, measured with the Brief Visuospatial Memory Test–Revised, and similarly in 2 of the 3 scores of verbal memory, measured with the Hopkins Verbal Learning Test–Revised.

#### Subjective Evaluations

Table 1 shows case 1’s rating of the technology at the end of every week of training (3 forms were filled). With the increase in game difficulty and session duration, his score for the question “The game controllers worked the way I wanted them to” progressively dropped from 4 to 3 to 2 (an average of 3, SD 1.00, out of 5). Scores for the question “The system was easy to use” saw a similar downward trend in the last and most difficult week of training (from 4 to 4 to 2). Case 1 indicated that he felt fatigued and scored low on the question “I was not fatigued at the end of the game therapy sessions,” with an average rating of 3.3, SD 0.58, out of 5. The highest scores were for the questions “The controllers made little noise” and “I had no muscle pain or discomfort,” both receiving an average score of 4.7, SD 0.58, out of 5. Despite some perceived difficulties with the controllers and his fatigue, case 1 gave an average score of 4, SD 0.00, out of 5 to the statements “I would encourage other participants to use it,” and “I liked the system overall.”

The attending OT for case 1 was equally positive, giving perfect scores to the statements “The controller provided good grasp training,” “The intensity of training was appropriate,” and “Overall, I am satisfied with the system.” The therapist was neutral (3 out of 5) when rating the ease of manually entering notes during the session, but 10 other statements were rated 4 out of 5.

#### Vital Signs

Over the 3-week training, case 1’s systolic and diastolic blood pressure values showed a decreasing trend at the end of each session. Readings dropped from 157/91 mm Hg after session 1 was completed to 126/74 mm Hg at the end of the last therapy session (Figure 5). Pulse increased slightly from 63 to 73 beats per minute for the same timeline.

### Case 2

#### Game Performance Outcomes

As seen in Figure 4, affected arm movement repetitions for case 2 started at 122 in session 1 and grew to a high of 957 total (left+right) arm repetitions per session in session 9. His grasp counts grew from 108 in session 1 to a maximum of 224 grasps (left and right hands combined) in session 10. Similarly, finger-extension counts increased from 62 in the first session to a high of 179 combined left- and right-hand extensions per session during the 3-week training. Case 2’s training intensity (repetitions per minute) grew by 362% for the arms and 150% for extensions per minute, but there was no increase in intensity for grasp training. Furthermore, there was a drop in grasp and finger-extension repetitions for the last 2 sessions. During that time, case 2 was tired; his last session had to be postponed by 1 day and ended up being shorter by one-third than initially planned.

Cognitive load increased for case 2 in proportion to the average game difficulty, which grew from 1.5 on average in the first session to a high of 2.9. Cognitive endurance, reflective of the length of play minutes per session, grew from 16 minutes in session 1 to 33 minutes of cognitive training per session toward the end of the 3-week training.

#### Motor Impairment

Motor impairment changes for case 2 on his affected left arm (also his dominant UE) are shown in Table 4. From before the training to after the training, his shoulder strength increased by 11.1 N for the anterior deltoid and by 6.6 N for the lateral deltoid, a vital outcome of the gravity-modulating feature of the BAC robotic rehabilitation table. Grasp strength improved from 96 N before the training to 111 N after the training. The 15-N gain was less than the MCID in the dominant arm of 49 N. With regard to 3-finger pinch strength, it grew from 12 N before the training to 14 N after the training (a 16% improvement). Affected arm active range of movement saw increases mainly in shoulder flexion (21° increase), elbow pronation (27° increase), and supination (33° increase). Case 2 had no change in his fingers’ range of movement, either in flexion or in extension, because they had normal range before the training.

#### Motor UE Function

Case 2’s UE function improved, with his Fugl-Meyer score increasing 10 points, equal to the MCID of 9-10 points for this measure. His speed of completing simulated ADLs, measured by the Jebsen Test of Hand Function, increased, resulting in a reduction in the task completion time of 25 seconds. This is better than the MCID of 20.8 seconds reduction for this measure. Bimanual ADL independence, measured by the Chedoke Arm and Hand Activity Inventory, improved by 14 points, well above the corresponding MCID of 6.3 points. On the standardized subjective UEFI 20-question self-report, the score improved 23 points for case 2, almost 3 times the UEFI MCID of 8 points.

#### Emotive and Cognitive Outcomes

Before the training, case 2’s BDI-II score of 5 indicated minimal depression. After the training, his depression severity had increased in the minimal range (score of 8). Case 2’s neurocognitive evaluations showed across-the-board gains in all his 6 tests of attention and processing speed. Executive function (NAB word generation raw score) increased from 4 before the training to 5 after the training. There were mixed outcomes in the tests for visual memory and similarly in those for verbal memory.

#### Subjective Evaluations

Case 2 gave an overall positive rating of the technology, averaging 3.95, SD 0.42, out of 5. His lowest average score of 3.7, SD 0.58, out of 5 was for the questions “Instructions given to me were useful,” “The game controllers worked the way I wanted them to,” “It was easy to put the controllers on and take them off,” “I was not fatigued by the end of the game therapy sessions,” and “There were few technical problems.” In addition to these above-average scores, case 2 responded very positively to the question “I would encourage other patients to use it,” which he scored at an average of 4.3, SD 0.58, out of 5. His highest average rating of 4.7, SD 0.58, out of 5 was for the statement “The controllers were easy to slide along the table.”

A different OT attending case 2’s training was equally positive, giving perfect scores to the statements “The controller provided good grasp training,” “The controller provided good finger extension training,” and “Patient did not appear to experience discomfort during exercises.” The lowest ratings of 2 out of 5 were for “It was easy to show the patient how to use the system,” “It was easy to set up and run the session,” and “There were few technical problems using the system.” The therapist agreed that they were satisfied with the system overall, with a rating of 4 out of 5.

#### Vital Signs

Case 2 started with very low blood pressure (80/52 mm Hg after session 1) and an elevated pulse of 81 beats per minute. Nonetheless, the attending physician and therapist had approved the participant for all activities, including the research study. Over the 3 weeks of experimental training, his systolic and diastolic blood pressure values increased steadily and his pulse rate improved. By the end of the last session, case 2’s blood pressure reading was 98/61 mm Hg and his pulse was 69 beats per minute.

## Discussion

### Principal Findings

The BAC rehabilitation system described here is an improvement over its BrightArm Duo predecessor in compactness (smaller footprint), the functionality of game controllers (added ability to detect finger extension as well as arm pronation and supination), and better tracking of UE 3D movement (tracking on and off the table vs only on the table for the BrightArm Duo). According to the subjective evaluation results, the OTs who were assisting the participants were satisfied with the technology (average score of 3.89, SD 0.62, out of 5) and successfully used it throughout the protocol. This indicates ease of learning of the new system because 2 different OTs were able to use it successfully.

Participants did not drop out or miss sessions. Their overall rating was positive (average 3.97, SD 0.03, out of 5), despite technical problems encountered, because this was the first clinical feasibility trial of the BAC system. Another possible explanation for the score stems from the exhaustion the participants may have experienced. The system received a positive rating in spite of game-based training intensity (up to 18-29 arm repetitions and 7-8 grasps every minute) and the fact that the participants were in the early subacute phase after stroke.

In this study, both participants improved from before the training to after the training in terms of their grasp strength (27% and 16%) and 3-finger pinch strength (31% and 17%). The improvements in grasp strength were below the MCID for subacute phase after stroke, which may be due to splitting of training time among finger flexion, extension, and forearm rotation movements instead of focusing on grasp alone.

By comparison, both participants had an improvement in UE function that was at or above the MCID for all four functional outcomes (Fugl-Meyer Assessment, Jebsen Test of Hand Function, Chedoke Arm and Hand Activity Inventory, and UEFI). The improvements in coordinated movements may explain the functional improvements seen in these individuals.

In the mood domain, the results were mixed, with depression severity reducing for case 1 (from minimal to normal) but increasing minimally (within normal variability) for case 2 (Table 5). In the cognitive domain of attention, both participants improved their auditory working memory as measured by the NAB digits backward subtest. In verbal memory, both improved in the recognition discrimination score (part of the Hopkins Verbal Learning Test–Revised). However, in the executive function domain, case 2 improved substantially in Trail Making Test B and in the NAB word generation subtest, whereas case 1 recorded negative gains on these tests. The combination of lower hand function, cognitive deficits, and depression may explain the overall lesser gains made by case 1.

Exit interviews were not conducted with the 2 cases, and their subjective evaluation form did not provide for comments on their experience. However, the therapists assisting the 2 participants took notes during their BAC sessions and on their feedback forms. The therapist assisting case 1 wrote that all movements needed assistance during week 1: “Patient needs verbal cues most of the time to squeeze/release and at times cues for which direction to move arm*.*” In week 2, this therapist noted as follows: “Different card categories is a nice element,” and in week 3, presumably with case 1 showing improvement, the therapist suggested, “Obstacles needed for Pick-and-Place to raise items over structure.” In the subjective BAC evaluation, this therapist wrote as follows: “Consider ‘symbols’ to encourage bimanual hand use. Larger Dum Circles for [week 3] difficulty.”

The therapist assisting case 2 wrote that during the first sessions, the movements needing assistance were forward reaching and lifting arm up: *“*[Case 2 needed] verbal/tactile cues to lift UE up when choosing game...voice cues to extend/grasp hand during Towers game*.*” During the last week’s sessions, this therapist noted, “Subject enjoyed to use both hands simultaneously for Pick-and-Place rather than unilaterally...[had] difficulty with Drums*.*” This observation provides a clue to the degree of functional improvement in case 2. Specifically, being able to simultaneously move the arms to reach targets while following 2 ideal trajectories implies ability to split attention and improved motor control.

### Limitations

This study included a limited number of participants (N=2), and the results cannot be generalized. This was due to a temporary drop in new admissions to the facility after the system had been installed, combined with the logistics of starting a follow-up randomized controlled trial (RCT) at another facility. This combination of factors limited the pool of potential candidates for the feasibility study described in this paper.

The BAC virtual rehabilitation component was added to the SOC rehabilitation that the participants were receiving as patients at an inpatient rehabilitation facility for patients in the subacute phase after stroke. During the 3 weeks of participation, the participants had 12 virtual rehabilitation sessions and 4 times as many SOC sessions (physical therapy, occupational therapy, and speech therapy). Thus, it is not possible to tell whether the VR intervention, SOC, or natural recovery was responsible for the improvements in their motor and cognitive domains. However, the improvements in gameplay were specific to BAC, as was the much higher training intensity (repetitions per minute), as opposed to SOC.

### Comparison With Prior Work

Other robotic rehabilitation tables exist in clinical use, such as the Bi-Manu-Track (HASOMED). Its shape resembles that of the BAC rehabilitation table in its center cutout, although the table is only horizontal, and bimanual training is for pronation and supination and finger flexion and extension. Although the Bi-Manu-Track does not have a VR component, its electrical actuators allow active and passive training of the impaired arms, whereas the BAC rehabilitation table only allows active UE training.

An open question within the rehabilitation robotics research community is whether robotic rehabilitation is superior to SOC of equal dosage and intensity when outcomes and costs are compared. One such study involved the Bi-Manu-Track as part of an RCT on 50 patients who were in the subacute phase after their first stroke [40]. The experimental group underwent training on several electrical devices, including Bi-Manu-Track, for 30 minutes, plus 30 minutes of individualized arm therapy, 5 days per week for 4 weeks. The control group had a matched duration and frequency of individualized arm therapy. The researchers reported no between-group differences in pre-post gains in the Fugl-Meyer Assessment, with the robot-assisted group therapy bearing half the cost of individualized arm therapy.

Another open question is whether game-based training *added to* SOC UE rehabilitation for patients in the subacute phase after stroke will produce higher outcomes than SOC alone. An RCT conducted by Wang et al [41] involved individuals who averaged 7.5 weeks after stroke and were assigned equally to an experimental group (n=13) and a control group (n=13). Each group received daily sessions of occupational therapy for 45 minutes, 5 days per week for 4 weeks. The experimental group received *additional* daily gaming sessions for 45 minutes each over the same duration, whereas the control group received a second occupational therapy session of equal length each day. Once the 4 weeks of experimental intervention were completed, all participants continued with one 45-minute session of standard occupational therapy, 5 days per week for 4 weeks. The pre-post outcome comparison using the Wolf Motor Function Test [42] showed a higher quality score for the experimental group, and the Wolf Motor Function Test time for the experimental group was significantly shorter than that for controls.

In a more recent study on patients in the acute and early subacute phase after stroke [43], researchers reported on an RCT where 7 participants had SOC (occupational therapy, physical therapy, and speech therapy) plus 8 sessions (1 hour each) of UE VR and robotics training. The control group consisted of 6 participants who received only their SOC rehabilitation. The pre-post comparison showed significantly larger gains on the Fugl-Meyer Assessment and the wrist active range of motion for the experimental group than for the control group. This study supports the belief that adding robotic and VR rehabilitation to SOC benefits patients early after a stroke. Although the BAC case study presented here did not compare with SOC alone, experimental training was nonetheless beneficial to the 2 participants.

### Conclusions

The feasibility case study presented here is the first clinical trial of the novel BAC system. Experimental training could be administered easily by OTs at an inpatient rehabilitation facility for patients in the subacute phase after stroke and benefited the 2 participants. To better determine the effect of added BAC training on SOC, an RCT involving participants in the acute and early subacute phase after stroke has been conducted. Data from this RCT are currently being analyzed, and results will be presented elsewhere.

In sum, the study contributes to the state of the science by illustrating that individuals with stroke are able to train on an integrative rehabilitation system with gains in UE motor function for different levels of severity of motor deficits. Another key contribution to the field is the limited gain noted in mood and cognition with training on the integrative system, which indicates that the training protocol with and without SOC needs to be re-examined. The responders and nonresponders to technology-based rehabilitation training systems need to be identified based on severity of deficit.

Another important finding is that participants liked the BAC system and would recommend it to others, with overall rating of their experience at 79% (3.95 out of 5). This is remarkable in view of the relative novelty of the system to, and high technology use by, older adults. This study supports an increasing body of evidence that shows older adults as being accepting of advanced technology in rehabilitation as long as the technology is intuitive to use [44-46].

## Abbreviations

ADL: activity of daily living
BAC: BrightArm Compact
BBG: BrightBrainer Grasp
BDI-II: Beck Depression Inventory, Second Edition
IR: infrared
MCID: minimal clinically important difference
NAB: neuropsychological assessment battery
OT: occupational therapist
RCT: randomized controlled trial
SOC: standard of care
UE: upper extremity
UEFI: Upper Extremity Functional Index
VR: virtual reality
